# Childhood mortality, intra-household bargaining power and fertility preferences among women in Ghana

**DOI:** 10.1186/s12978-019-0798-2

**Published:** 2019-09-10

**Authors:** Jacob Novignon, Nadege Gbetoton Djossou, Ulrika Enemark

**Affiliations:** 1 Department of Economics, Kwame Nkrumah University of Science and Technology, Kumasi, Ghana; 2 Department of Economics, University of Abomey-Calavi, Abomey-Calavi, Benin; 3 Department of Public Health, Aarhus University, Aarhus, Denmark

**Keywords:** Fertility, child mortality, Bargaining power, Ghana

## Abstract

**Background:**

Continuing population growth could be detrimental for social and economic wellbeing. Understanding the factors that influence family planning decisions will be important for policy. This paper examines the effect of childhood mortality and women’s bargaining power on family planning decisions.

**Methods:**

Data was from the 2014 Ghana Demographic and Health Survey (DHS). A sample of 3313 women in their reproductive age were included in this study. We created variables on women’s exposure to and experience of child mortality risks. Three different indicators of women’s bargaining power in the household were also used. Probit models were estimated in accordance with the nature of the dependent variable.

**Results:**

Results from the probit models suggest that child mortality has a positive association with higher fertility preference. Also, child mortality risks and woman’s bargaining power play important roles in a woman’s fertility choices in Ghana. Women with higher bargaining power were likely to prefer fewer children in the face of child mortality risks, compared to women with lower bargaining power.

**Conclusion:**

In addition to public sensitization campaigns on the dangers of high fertility and use of contraceptives, the findings of this study emphasize the need to focus on reducing child mortality and improving women bargaining power in developing countries.

## Plain English summary

This paper examines the relationship between childhood mortality, bargaining power and family planning decisions. Data was from the Ghana Demographic and Health Survey (DHS). We created variables on women’s exposure to and experience of child mortality risks. Three different indicators of women’s bargaining power in the household were also used. Results from probit models suggest that child mortality has a positive association with higher fertility preference. Also, child mortality risks and woman’s bargaining power play important roles in a woman’s fertility choice in Ghana. Women with higher bargaining power were likely to prefer fewer children in the face of child mortality risks, compared to women with lower bargaining power. In addition to public sensitization campaigns on the dangers of high fertility and use of contraceptives, the findings of this study emphasize the need to focus on reducing child mortality and improving women bargaining power in developing countries.

## Background

Curbing rapid population upsurge has become important to policy makers all over the world. This is particularly relevant in developing countries where rapid population growth could result in increased poverty level and reduced wellbeing, in general. On a broader scale, continuing population growth could hinder a country’s economic performance as this raises pressure on already limited public infrastructure and places extra strain on national government budgets [1]. The nexus between population growth, household welfare and economic performance is made clear in the quantity-quality tradeoff theory by Becker and Lewis [3]; Becker and Tomes [4]; Becker and Barro [2] and Willis [26]. The theory posits that there exists a trade-off between having more children and raising quality children. They hypothesize that households with lower family size are more likely to have higher quality of life relative to households with larger family size. For instance, smaller households are more likely to provide better education and health care to its members than larger households, ceteris paribus.

Moreover, households with relatively smaller family sizes are more likely to benefit from demographic dividends. For instance, households with smaller family sizes have less pressure on scarce resources which could be invested in economic ventures and family welfare. Such families are also likely to have higher per-capita income than larger families [19]. This suggests that when population growth is not controlled it could have negative implications for the economy as a whole and the welfare of individual households within the economy. Aside its economic implications, increased fertility rates could have devastating effect on maternal and child health [10, 18].

Attempts to control population growth have over the years focused on meeting contraceptive needs of the population and improving public education on the awareness and proper use of modern contraceptives. In Ghana, population reduction policies over the years include the Contraceptive Social Marketing (CSM) project (1987–1990), the Ghana Family Planning and Health Programme (FPHP) (1990–1996) and the Ghana Population and AIDS Project (GHANAPA) (1996–2000) [14]. A more recent policy effort targets reducing fertility rate to 3.0 by the year 2020 as well as reducing population growth to 1.5% by the same year. While the early policies resulted in a marked decline in fertility by the 1990s, recent data suggest a slight rise in the fertility rate. The total fertility rate (TFR) declined from 6.4 births per woman in 1988 to 5.2 in 1993 then dropped further to 4.0 in 2008. However, in 2014, TFR increased marginally to 4.2 births per woman. This raises concerns about progress towards achieving the 3.0 target [11].

An important step to achieve the set population growth target is to understand the practical determinants, not only of fertility rate but also fertility preferences among women and couples, by extension. Several socioeconomic and demographic factors have been identified in previous empirical studies. Some of these include the number of children ever born, education [24] and infant mortality [21]. Other factors include maternal age, wealth status of the household, and engagement in economic activities, among others [5, 16, 17, 22].

In developing countries such as Ghana, infant and child mortality continue to significantly influence reproductive behavior among both men and women. This is largely due to the high rates of infant and child mortality in these countries [25]. It is therefore important that linkages between childhood mortality and reproductive behavior is well understood. Another relevant factor that has received minimal attention in the empirical literature is the nexus between women’s bargaining power and reproductive health behavior. Again, this is particularly important in developing countries where women are often considered to have no or little influence on fertility or fertility preferences. While a clear understanding of these relationships will be crucial for effective targeting and policy direction, very little evidence exists in developing countries such as Ghana.

### Conceptual framework

The conceptual linkages between childhood mortality and fertility preferences hinges on two strands of theoretical literature. These theoretical predictions seek to explain the behavioral responses of individuals or households to childhood mortality [13]. The first hypothesis posits that households choose to have additional children to replace dead children so that marginal changes in net fertility due to child mortality is zero. This implies that households have targeted numbers of children and that reductions below this target generates disutility. The hypothesis is known in the literature as the replacement hypothesis [7, 13]. The second hypothesis assumes that households have or prefer to have more children as precaution when uncertainties about child survival are high. This is particularly pertinent in developing countries where child mortality remains a major public policy concern. This implies that families facing relatively higher child mortality risk will adapt their fertility behavior accordingly. This hypothesis is known as the child survival hypothesis or hoarding motive [6]. In other studies, this hypothesis has been referred to as ‘anticipatory effect’ [13] or insurance effect [7]. While the former hypothesis is predicted to impact only total fertility positively, the latter is expected to affect both total and net fertility positively. In this study, we hypothesize that child mortality and related uncertainties have positive impact on fertility preferences. We further posit that women’s intra-household bargaining power has a role to play in the child mortality – fertility preference nexus. Against this backdrop, our study deviates from existing studies mainly in our focus on fertility preferences instead of actual fertility. While we consider fertility preference to mean the number of children a woman wishes or desires to have, we define fertility as the actual number of children a woman already has. To this end we sought to provide answers to two research questions; (i) do childhood mortality and women’s bargaining power affect fertility preferences? (ii) are there interactive effects between child mortality, women’s bargaining power and fertility preferences?

### Empirical review

The literature on fertility preferences and its determinants have evolved over the years. While some researchers focus on just preferences, others have looked at stability in these preferences over time. As indicated earlier, several socioeconomic and demographic variables have been identified in these studies. Broadly, these factors encompass both gender characteristics and household level factors. For instance, Nyarko et al. [21] investigated the link between male child loss and subsequent fertility in Ghana using the 1993 Ghana Demographic and Health Survey (DHS). The authors sought to find out if there is preference for male children. Using parity progression ratios and time hazard models, the findings of the study suggest that while the death of an infant induced mothers to have another child, the death of a male child reduced the birth interval greatly. Similarly, Tawiah [24] also used the 1993 Ghana DHS data to identify socioeconomic and demographic factors that influence child preferences with specific emphasis on male child preference. The findings showed that male child preference was significantly associated with level of education, region of residence, experience of child loss and religion.

In recent years, there has been growing interest in the relationship between child mortality and fertility in developing countries. Bousmah [6] used micro level data from a demographic and health surveillance system in a rural community in Senegal to explore the relationship between child mortality and fertility. Results from the standard Poisson regression model confirmed the child survival hypothesis with a positive effect of child mortality on both total and net fertility. In a related study, Bousmah [7] used longitudinal data from the same surveillance system in Senegal and found that both the replacement hypothesis and hoarding motives were true in the case of Senegal. The author confirmed that morbidity and mortality from malaria jointly affected subsequent fertility choices positively. Following the Indian Ocean Tsunami in 2004, Nobles et al. [20] found that women who lost one or more children are likely to bear additional children.

In a cross-country study, Canning et al. [8] used data from 46 low and middle-income countries to estimate the relationship between child mortality and fertility. It was evident from their study that reducing child mortality will likely reduce the number of children born but increase the number of surviving children and therefore lead to a rising population growth. The authors also found that where an individual’s fertility choices affect the fertility choices of others (they call this interdependent fertility preferences), the net effect of child mortality on population growth rate is zero. Sennott and Yeatman [23] examined the level and direction of changes in fertility preferences among women. Using panel data from Malawi and multinomial logit model, the authors found that having a child, entering a serious relationship and changes in finances of the household were associated with changes in the level and direction of fertility preferences. In a more qualitative analysis, DeRose et al. [9] investigated the role of perceptions of power in reproductive conflict in a woman’s fertility desires. Using focus group discussions among young Ghanaian men and women, the findings showed that young women’s expected influence were limited to situations where their fertility desires conform to normative expectations. In another study to test the stability in individual fertility preference over time, Kodzi et al. [15] used panel data from Ghana. The authors found that about 20% of respondents changed their fertility preference over time.

The discussions of the literature so far suggest previous studies mostly focused on the relationship between child mortality and fertility. Studies that analyzed fertility preference failed to account for the effect of child mortality. Moreover, the potential role of women’s bargaining power in this relationship has been absent in the literature. We therefore contribute to existing studies in developing countries in this regard. We explored the relationship between child mortality, bargaining power and fertility preferences among Ghanaian women in their reproductive age. We also estimated the interactive effect of these relationship.

## Methods

### Data and variable description

The study relied on data from the 2014 Demographic and Health Survey (DHS) in Ghana. This is the most recent DHS in Ghana and is a nationally representative cross-sectional data set collected on men, women and children. The survey collects information on individual’s demographic and health characteristics as well as some social and economic variables. Specifically, the survey collects information on fertility and fertility preferences, household decision making, childhood mortality, maternal and child health, among others. The sample design was conducted at two stages; the first stages selected clusters consisting of enumeration areas following the 2010 Population and Housing Census. A total of 427 clusters were selected with 216 in urban and 211 in rural areas. In the second stage households were systematically sampled. A total sample size of 12,831 households were selected [11]. A total of 9396 eligible women were interviewed. In this study, we focus on mothers and this reduced the sample to 3313 women who have given birth to at least one child.

Three different indicators of women’s fertility preferences are used as dependent variables. The first is a dummy variable ‘More children’ that captures a woman’s desire to have more children. The variable takes the value of 1 if a woman desires more children and 0 otherwise. While the variable captures fertility preference, it is limited in the sense that it does not account for the number of children a woman currently has. For instance, a woman who currently has one child and desires more children will be treated equally as a woman with four children and desires more. To account for this limitation, we created a dummy variable ‘Net desire’ that takes the value of 1 if a woman currently has more than three living children and still desires more, otherwise 0. The choice of three children is justified by the global average fertility rate for lower middle-income countries such as Ghana. This variable is used as the second indicator of fertility preference. The third and final indicator of fertility preference follows Handa [13]. We created a dummy variable ‘Extra fertility’ capturing the difference between a woman’s ideal and actual number of children. Women whose ideal number of children is less than the actual number of children are classified as having higher fertility preference and take the value of 1, otherwise 0. The DHS asks women to state their ideal number of children if they had the chance to start all over again. This variable was explored in addition to the actual number of children to generate the third fertility preference dummy variable. Using these three variables also allow us to test the robustness of our findings.

Two main independent variables are used to test the hypothesis under investigation. These are the child mortality and women’s bargaining power variables. We used three different measures of child mortality: (i) predicted child mortality risk (ii) experienced child mortality dummy and (ii) child mortality ratio. The predicted values from the regression is obtained and used as proxy for child mortality risk. The predicted values measure the odds of childhood death in a given cluster. We hypothesize that women with high exposure to child mortality within the cluster will have higher fertility preference in line with the ‘insurance effect’. The second child mortality variable is a dummy variable that takes the value of 1 if a woman has previously experienced child death. Finally, we compute a child mortality ratio which equals to number of child deaths divided by total children born. The last two child mortality indicators are measured at the individual level while the first was measured at the cluster level.

The DHS includes questions on decision making with respect to health care, contraceptive use, and household purchases. There were also questions on women’s ownership of assets. We used these variables to compute a bargaining power index with principal component analysis (PCA). The PCA procedure allows us to determine which of the bargaining power components is relevant. It turned out that women decision making on health and purchases were important for the bargaining power index with eigen values above one. We also hypothesize that there exists an interactive effect between child mortality, women bargaining power and fertility preference. We therefore created various interaction variables that seek to find out if the impact of child mortality on fertility preference differs for women with better bargaining power.

A number of other variables were included in the analysis as controls in line with literature. At the individual level, the control variables included age of the woman, place of residence (rural/urban), education, National Health Insurance Scheme (NHIS) coverage and employment status. For married women, characteristics of the husband were also included. These include education of the husband and whether or not he is a polygamous husband. The general economic situation of the household was also controlled for using the asset-based wealth quintiles available in the data set.

### Empirical specification

The empirical model for the study is presented in eq. (1). The model specifies the relationship between the various child mortality variables, women’s bargaining power and fertility preference.

1 $$ {f}_i={\alpha}_i+{\beta}_1{z}_i+{\beta}_2{v}_i+\sum \limits_{n=3}^N{\beta}_n{x}_i+{\varepsilon}_i $$

Where *f*_*i*_ captures fertility preference of the *i*^*th*^ woman, *z*_*i*_ is childhood mortality indicator, *v*_*i*_ is women’s bargaining power, *x*_*i*_ is a vector of socioeconomic and demographic characteristics and ε is the error term. Several specifications of the model were estimated using the different indicators of the variables. As mentioned earlier this was necessary to ensure the estimates are robust measures of the variables of interest.

To capture the interaction effect, eq. (1) was modified to incorporate the interaction terms. The extended specification is presented in eq. (2).

2 $$ {f}_i={\alpha}_i+{\beta}_1{z}_i+{\beta}_2{v}_i+{\beta}_3z\ast {v}_i+\sum \limits_{n=4}^N{\beta}_n{x}_i+{\varepsilon}_i $$

The fourth term in eq. (2) represents the interaction between child mortality and bargaining power. The significance and sign of this term allows us to determine whether the impact and direction of child mortality on fertility preferences depend on a woman’s bargaining power. All other variables in the model remain the same. Again, various specifications of the model were estimated using different indicators for the variables of interest.

Given that all the dependent variables are dummy variables, we used binary response econometric models for estimation. Specifically, we used the Probit model which assumes a cumulative normal distribution function [12].

## Results

We begin the results section with a presentation of descriptive statistics of variables included in the analysis (see Table 1). This is followed by a presentation of estimation results of the association between fertility preference, child mortality and women’s bargaining power. Within each regression table, the results are presented for the three measures of child mortality. For each type of child mortality measure, we present the result for two different models. The difference between these two models is that the second one includes interaction between child mortality and women’s bargaining power while the first doesn’t include the interactions.

**Table 1:** Descriptive statistics

Variable name	Mean	Standard deviation	Min	Max
Fertility preference (more children)	0.600	0.490	0	1
Fertility preference (net desire)	0.126	0.332	0	1
Fertility preference (extra fertility)	0.481	0.500	0	1
Women’s bargaining (health)	0.795	0.404	0	1
Women’s bargaining (purchases)	0.752	0.432	0	1
Women’s bargaining power index (dummy)	50.84	0.50	0	1
Child mortality	0.24	0.42	0	1
Child mortality risk	0.25	0.14	0	0.63
Child mortality ratio	0.07	0.15	0	1
Urban location	0.465	0.499	0	1
Age	33.750	7.556	15	49
Woman’s Education				
None	0.337	0.473	0	1
Primary	0.186	0.389	0	1
Secondary	0.426	0.495	0	1
Tertiary	0.055	0.227	0	1
Husband’s Education				
None	0.264	0.441	0	1
Primary	0.114	0.318	0	1
Secondary	0.501	0.500	0	1
Tertiary	0.112	0.315	0	1

Source: Authors’ computation

### Descriptive statistics

Table 1 presents summary statistics of variables included in our analysis. The statistics show that about 60% of women reported desire for more children, irrespective of current number of children. However, when fertility preference was measured using the difference between desired and actual number of children (net desire), about 13% of women were classified as preferring more children. That is, about 13% of women currently have at least 3 children and still desire more. The third indicator of fertility preference shows about 48% of women preferring more children. This implies that 48% of women gave birth to more children than they desired. Average fertility in the sample was 3.6 children. It was also evident that a majority of the women in the sample participated in household decision making with regards to health (79.5%) and purchases (75.2%). However, when we consider the composite index for bargaining power, we found that 50.84% of women have some bargaining power within the household.

Moreover, Table 1 suggests that 24% of women have experienced child mortality whereas 25% of them are exposed to child mortality risk at the cluster level. On average, the child mortality ratio for each woman is equal to 0.07. Average age among women included in the sample was 33 years. Majority of women (42.6%) and husbands (50.1%) in the sample had completed secondary education.

### Preference for more children and child mortality

Table 2 presents results of the effect of child mortality and women’s bargaining power on their preference for more children in Ghana. The fertility preference is measured here as a dummy variable that takes the value of one if a mother prefers to have more children. The results show a positive and statistically significant (at 1%) relationship between the three measures of child mortality risk and the desire of a mother to have more children. The results are largely consistent across all models. This suggests that mothers who are relatively more exposed to child mortality or experienced child mortality themselves are likely to desire more children. Also, women’s desire to have more children increases with the ratio of child mortality which equals to the number of child deaths divided by total children born.

**Table 2:** Fertility preferences (more children) and child mortality

	Child mortality risk		Child mortality (Dummy)		Child mortality rate	
	Model 1	Model 2	Model 1	Model 2	Model 1	Model 2
CM (Risk, Dummy, rate)	1.344***	1.346***	0.642***	0.649***	2.368***	2.388***
	(0.227)	(0.227)	(0.074)	(0.075)	(0.261)	(0.267)
WBP (Index)	− 0.085***	− 0.073*	− 0.095***	− 0.082***	− 0.096***	− 0.090***
	(0.020)	(0.040)	(0.021)	(0.023)	(0.021)	(0.023)
Index*CM	–	−0.049	–	− 0.055	–	− 0.107
	–	(0.142)	–	(0.046)	–	(0.190)
Urban location	0.012	0.012	−0.045	− 0.048	− 0.046	− 0.048
	(0.073)	(0.073)	(0.074)	(0.073)	(0.074)	(0.074)
Age	−0.044***	−0.044***	− 0.048***	− 0.048***	− 0.049***	− 0.049***
	(0.006)	(0.006)	(0.007)	(0.007)	(0.007)	(0.007)
Woman’s education						
Primary	−0.271***	− 0.270***	− 0.266***	− 0.267***	− 0.270***	−0.271***
	(0.093)	(0.093)	(0.093)	(0.094)	(0.094)	(0.094)
Secondary	−0.372***	− 0.372***	− 0.372***	− 0.371***	− 0.370***	−0.369***
	(0.088)	(0.088)	(0.087)	(0.087)	(0.088)	(0.088)
Higher	−0.636***	− 0.637***	− 0.620***	− 0.619***	− 0.618***	−0.617***
	(0.151)	(0.151)	(0.154)	(0.154)	(0.156)	(0.156)
Husband’s education						
Primary	−0.378***	− 0.376***	− 0.385***	− 0.381***	− 0.394***	− 0.392***
	(0.111)	(0.111)	(0.110)	(0.110)	(0.109)	(0.109)
Secondary	−0.470***	−0.469***	− 0.467***	−0.464***	− 0.481***	−0.480***
	(0.095)	(0.095)	(0.094)	(0.094)	(0.094)	(0.094)
Higher	−0.340***	− 0.339***	− 0.333***	− 0.332***	− 0.352***	−0.352***
	(0.123)	(0.123)	(0.124)	(0.124)	(0.125)	(0.125)
Household Wealth status						
Poor	−0.186**	− 0.184**	− 0.242***	− 0.238***	−0.246***	− 0.244***
	(0.092)	(0.092)	(0.091)	(0.091)	(0.091)	(0.091)
Middle	−0.155	−0.155	− 0.234**	−0.229**	− 0.229**	−0.227**
	(0.100)	(0.100)	(0.101)	(0.101)	(0.102)	(0.102)
Rich	−0.205*	−0.203*	− 0.290**	−0.286**	− 0.291**	−0.290**
	(0.114)	(0.114)	(0.116)	(0.116)	(0.116)	(0.116)
Richest	−0.165	−0.164	−0.272**	− 0.267**	−0.282**	− 0.280**
	(0.127)	(0.127)	(0.129)	(0.129)	(0.130)	(0.131)
Husband’s preference	−0.043	−0.044	− 0.056	−0.060	− 0.057	−0.058
	(0.062)	(0.062)	(0.062)	(0.062)	(0.062)	(0.063)
Number of children	−0.397***	−0.397***	− 0.428***	−0.429***	− 0.413***	−0.414***
	(0.022)	(0.022)	(0.025)	(0.025)	(0.024)	(0.024)
NHIS coverage	0.053	0.052	0.055	0.055	0.055	0.054
	(0.060)	(0.060)	(0.060)	(0.060)	(0.061)	(0.061)
Husband’s age	−0.005	−0.005	−0.004	−0.004	− 0.004	−0.004
	(0.004)	(0.004)	(0.004)	(0.004)	(0.005)	(0.005)
Self employed	−0.006	−0.005	0.002	0.005	−0.005	−0.003
	(0.070)	(0.070)	(0.071)	(0.071)	(0.072)	(0.072)
Polygamous husband	0.057	0.058	0.059	0.059	0.059	0.059
	(0.080)	(0.080)	(0.080)	(0.079)	(0.080)	(0.080)
Constant	3.795***	3.794***	4.262***	4.255***	4.277***	4.273***
	(0.206)	(0.206)	(0.202)	(0.202)	(0.204)	(0.204)
Pseudo R2	0.342	0.342	0.333	0.333	0.340	0.340
*N*	3255	3255	3072	3072	3072	3072

Source: Authors estimation

Model 2 includes interaction term

Robust standard errors in parentheses

*WBP* Women’s Bargaining Power

* *p* < 0.1 ** *p* < 0.05; *** *p* < 0.01

Furthermore, the women’s bargaining power index in the household is significantly and negatively related to fertility preferences across all model specifications. This implies that women with relatively stronger bargaining power are less likely to desire more children. The interactive term is also negatively associated with women’s fertility preference. This suggests that while women’s exposure to child mortality may increase their probability of desiring more children, this depends on the woman’s bargaining power. Women with relatively better bargaining power within the household were less likely to desire more children even when they faced high child mortality. The relationship, however, did not show any statistical significance.

For the different model specifications, the results suggest that, older woman are less likely to desire more children. Also, women’s preference for more children decrease as their education level increases. However, the place of residence, women’s employment status, husband’s fertility preference and polygamous status do not have any significant effect on women’s desire for more children.

### Net desire for more children and child mortality

In Table 3, child mortality measures were estimated on the second indicator of fertility preference which we referred as net desire. Across all model specifications, there was evidence of a significant and positive relationship between the three measures of child mortality and women’s net desire for fertility. Women’s bargaining power index is again negatively related to fertility preference, even though there is no significant evidence that the relationship between child mortality risk and fertility preference depends on women’s bargaining power when the model includes interactive term. As in the results in Table 2, the interactive term is also negatively associated with women’s net desire for more children, even though the estimates are not significant.

**Table 3:** Fertility preferences (Net Desire), child mortality and women’s BP in Ghana

	Child mortality risk		Child mortality (Dummy)		Child mortality rate	
	Model 1	Model 2	Model 1	Model 2	Model 1	Model 2
CM (Risk, Dummy, rate)	0.751***	0.779***	0.563***	0.589***	2.072***	2.118***
	(0.222)	(0.225)	(0.077)	(0.080)	(0.277)	(0.293)
WBP (Index)	−0.054***	− 0.010	− 0.063***	− 0.047**	− 0.062***	− 0.056**
	(0.021)	(0.042)	(0.021)	(0.023)	(0.021)	(0.023)
Index*CM		−0.176		− 0.078		− 0.144
		(0.151)		(0.052)		(0.191)
Urban location	0.021	0.021	−0.015	−0.018	− 0.014	− 0.015
	(0.070)	(0.070)	(0.072)	(0.072)	(0.071)	(0.071)
Age	−0.016**	− 0.016**	− 0.017***	− 0.018***	− 0.018***	− 0.018***
	(0.006)	(0.006)	(0.007)	(0.007)	(0.007)	(0.007)
Woman’s education						
Primary	−0.016	− 0.012	− 0.003	0.003	− 0.003	− 0.001
	(0.093)	(0.093)	(0.094)	(0.094)	(0.094)	(0.094)
Secondary	−0.140	− 0.138	− 0.134	−0.132	− 0.134	−0.132
	(0.086)	(0.086)	(0.086)	(0.086)	(0.086)	(0.086)
Higher	−0.336**	− 0.340**	− 0.374**	− 0.372**	−0.371**	− 0.370**
	(0.155)	(0.155)	(0.159)	(0.159)	(0.160)	(0.160)
Husband’s education						
Primary	−0.095	− 0.090	− 0.075	− 0.073	−0.085	− 0.084
	(0.107)	(0.108)	(0.106)	(0.106)	(0.106)	(0.106)
Secondary	−0.079	− 0.075	− 0.054	− 0.051	− 0.067	−0.066
	(0.088)	(0.088)	(0.088)	(0.088)	(0.088)	(0.088)
Higher	−0.183	− 0.181	− 0.172	− 0.173	−0.192	− 0.194
	(0.118)	(0.118)	(0.119)	(0.118)	(0.119)	(0.119)
Household Wealth status						
Poor	−0.042	− 0.038	− 0.071	− 0.066	−0.076	− 0.074
	(0.088)	(0.088)	(0.089)	(0.089)	(0.088)	(0.088)
Middle	−0.170*	− 0.167*	− 0.225**	− 0.221**	−0.219**	− 0.218**
	(0.097)	(0.097)	(0.098)	(0.098)	(0.097)	(0.097)
Rich	−0.254**	−0.248**	− 0.309***	−0.305***	− 0.308***	−0.305***
	(0.112)	(0.112)	(0.113)	(0.113)	(0.113)	(0.113)
Richest	−0.019	−0.018	−0.090	− 0.085	−0.092	− 0.090
	(0.132)	(0.132)	(0.133)	(0.133)	(0.132)	(0.132)
Husband’s preference	−0.052	− 0.057	−0.060	− 0.064	−0.055	− 0.057
	(0.061)	(0.061)	(0.062)	(0.062)	(0.062)	(0.062)
Number of children	−0.152***	−0.152***	− 0.179***	−0.180***	− 0.167***	−0.167***
	(0.015)	(0.015)	(0.017)	(0.017)	(0.017)	(0.017)
NHIS coverage	−0.058	−0.060	−0.051	− 0.049	−0.051	− 0.050
	(0.060)	(0.060)	(0.061)	(0.061)	(0.061)	(0.061)
Husband’s age	0.003	0.003	0.003	0.003	0.003	0.003
	(0.005)	(0.005)	(0.005)	(0.005)	(0.005)	(0.005)
Self employed	−0.060	−0.058	−0.042	− 0.039	−0.045	− 0.044
	(0.073)	(0.074)	(0.075)	(0.075)	(0.076)	(0.076)
Polygamous husband	0.135*	0.135*	0.136*	0.133*	0.133*	0.132*
	(0.079)	(0.079)	(0.080)	(0.080)	(0.080)	(0.080)
Constant	2.257***	2.246***	2.464***	2.452***	2.453***	2.447***
	(0.180)	(0.181)	(0.171)	(0.171)	(0.173)	(0.172)
Pseudo R2	0.078	0.078	0.083	0.084	0.086	0.086
*N*	3600	3600	3398	3398	3398	3398

Source: Authors estimation

Model 2 includes interaction term

Robust standard errors in parentheses

*WBP* Women’s Bargaining Power

* *p* < 0.1 ** *p* < 0.05; *** *p* < 0.01

By contrast to the models with preference for more children (Table 1), we find that the husband polygamous status has a positive and significant (at 10%) effect on women’s net desire for children. The age and educational status of the woman both showed negative relationship with net desire for children. This suggests that older and more educated women are likely to prefer relatively less children compared to the young and uneducated.

### Extra fertility and child mortality

The estimates of the relationship between women’s extra fertility preference, child mortality measures are presented in Table 4. Similar to the previous results, we found a strong positive relationship between the different measures of child mortality and extra fertility preference. The results were also consistent across model specifications. The results imply that women who experienced child mortality were likely to have more children than they had planned (Extra fertility). Again, the results show a consistent negative relationship between women’s bargaining power index and extra preference for children. Models with the interactive terms also show negative association with fertility preference even though statistical significance was limited to one interactive term at 10% statistical level.

**Table 4:** Fertility preferences (Extra fertility), child mortality a in Ghana

	Child mortality risk		Child mortality (Dummy)		Child mortality rate	
	Model 1	Model 2	Model 1	Model 2	Model 1	Model 2
CM (Risk, Dummy, rate)	1.236***	1.231***	0.539***	0.547***	1.732***	1.802***
	(0.201)	(0.201)	(0.065)	(0.066)	(0.220)	(0.219)
WBP (Index)	−0.073***	− 0.029	− 0.084***	− 0.068***	− 0.086***	− 0.073***
	(0.018)	(0.037)	(0.018)	(0.021)	(0.018)	(0.020)
Index*CM	–	−0.176	–	− 0.074*	–	− 0.235
	–	(0.127)	–	(0.040)	–	(0.146)
Urban location	0.055	0.053	−0.018	− 0.022	− 0.016	−0.019
	(0.066)	(0.066)	(0.068)	(0.068)	(0.068)	(0.068)
Age	−0.027***	− 0.027***	− 0.029***	− 0.030***	− 0.030***	−0.030***
	(0.006)	(0.006)	(0.006)	(0.006)	(0.006)	(0.006)
Woman’s education						
Primary	−0.157**	−0.154**	− 0.150*	− 0.150*	− 0.152*	− 0.154*
	(0.077)	(0.077)	(0.079)	(0.080)	(0.079)	(0.080)
Secondary	−0.331***	− 0.329***	− 0.328***	− 0.328***	− 0.325***	− 0.324***
	(0.073)	(0.073)	(0.075)	(0.075)	(0.075)	(0.076)
Higher	−0.746***	−0.749***	− 0.733***	− 0.731***	− 0.730***	− 0.726***
	(0.150)	(0.150)	(0.157)	(0.158)	(0.158)	(0.158)
Husband’s education						
Primary	− 0.165*	− 0.160*	− 0.204**	− 0.201**	− 0.212**	− 0.210**
	(0.088)	(0.088)	(0.090)	(0.090)	(0.090)	(0.090)
Secondary	−0.441***	− 0.437***	− 0.432***	− 0.429***	− 0.446***	− 0.444***
	(0.076)	(0.076)	(0.078)	(0.078)	(0.078)	(0.078)
Higher	−0.470***	− 0.468***	− 0.444***	− 0.445***	− 0.467***	−0.468***
	(0.109)	(0.109)	(0.113)	(0.113)	(0.114)	(0.114)
Household Wealth status						
Poor	−0.129*	−0.124	−0.197**	− 0.191**	− 0.195**	− 0.191**
	(0.076)	(0.076)	(0.077)	(0.078)	(0.077)	(0.077)
Middle	−0.115	− 0.112	− 0.210**	−0.205**	− 0.206**	−0.202**
	(0.086)	(0.086)	(0.088)	(0.088)	(0.088)	(0.089)
Rich	−0.337***	−0.331***	− 0.446***	−0.443***	− 0.446***	−0.444***
	(0.102)	(0.103)	(0.105)	(0.105)	(0.105)	(0.105)
Richest	−0.345***	−0.341***	− 0.434***	−0.427***	− 0.441***	−0.437***
	(0.116)	(0.116)	(0.119)	(0.119)	(0.120)	(0.120)
Husband’s preference	−0.088	−0.094*	− 0.079	−0.085	− 0.077	−0.082
	(0.056)	(0.056)	(0.057)	(0.057)	(0.057)	(0.057)
Number of children	−0.211***	−0.211***	− 0.268***	−0.270***	− 0.248***	−0.251***
	(0.017)	(0.017)	(0.019)	(0.019)	(0.019)	(0.019)
NHIS coverage	0.132**	0.129**	0.110**	0.109**	0.114**	0.113**
	(0.054)	(0.054)	(0.056)	(0.056)	(0.056)	(0.056)
Husband’s age	−0.007*	−0.007*	−0.006	−0.006	− 0.006	−0.006
	(0.004)	(0.004)	(0.004)	(0.004)	(0.004)	(0.004)
Self employed	0.137**	0.139**	0.127*	0.131**	0.121*	0.125*
	(0.063)	(0.063)	(0.066)	(0.066)	(0.066)	(0.066)
Polygamous husband	0.077	0.077	0.084	0.085	0.084	0.085
	(0.069)	(0.069)	(0.071)	(0.071)	(0.071)	(0.071)
Constant	2.054***	2.054***	2.624***	2.620***	2.609***	2.606***
	(0.168)	(0.168)	(0.163)	(0.163)	(0.163)	(0.163)
Pseudo R2	0.187	0.187	0.199	0.199	0.204	0.205
*N*	3255	3255	3072	3072	3072	3072

Source: Authors estimation

Model 2 includes interaction term

Robust standard errors in parentheses

*WBP* Women’s Bargaining Power

* *p* < 0.1 ** *p* < 0.05; *** *p* < 0.01

Moreover, aside the age and education of the woman, the results also show that NHIS coverage and self-employment have positive and consistent effect on the desire to have extra children.

## Discussions

The study set out to examine the relationship between child mortality and fertility preferences among Ghanaian women in their reproductive age. The study was motivated by the relatively high fertility rate in Ghana and the potentially devastating impact on wellbeing and economy as a whole. Against this backdrop, understanding women’s fertility behaviour and how child mortality can affect it is crucial. We also hypothesized that a woman’s influence in the household could be relevant in this relationship. The data from the latest demographic and health survey was used for the analysis.

In general, the results suggest that child mortality (measured either in terms of a woman’s exposure or experience) has a positive association with fertility preference. This relationship was robust with consistency across model specifications and variable definitions. This result is also consistent with existing studies that sought to estimate similar relationships [6]. There was also significant evidence that suggest that aside child mortality, a woman’s bargaining power plays an important role in a woman’s fertility choices including the response to child mortality exposure or experience. We found that, in general, women with lower intra-household bargaining power were likely to prefer more children. There was also evidence that the effect of child mortality on fertility preference cannot be generalized for all women. We found from the interaction terms that, women with higher bargaining power were likely to prefer fewer children in the face of child mortality. This may be attributed to the fact that women with higher bargaining power are economically active and find more children as constraint to their activities. They are therefore likely to prefer fewer children.

The findings suggest that while previous studies have extensively identified various socioeconomic variables that may drive fertility upwards, the role of child mortality and women’s bargaining power cannot be over emphasized. Indeed, we observe that attempts to reduce child mortality and subsequently reduce the risk women face in this regard can change their perception about fertility and their choices thereof. In addition to public sensitization campaigns on the dangers of high fertility and use of contraceptives, the findings of this study emphasize the need to focus on reducing child mortality in developing countries and to sensitize women on the achievements made in reducing child mortality. While these findings underscore previous policies towards improving child health, it also reinforces current and future oriented policies. For instance, the first three of the recently adopted Sustainable Development Goals (SDGs) are directly targeted towards improving, among others, child health. Ensuring these goals are achieved, particularly in developing countries, could go a long way to control population growth. In addition to these, policies to promote women empowerment should also be encouraged. Like many other developing countries, Ghana continues to face significant gender gaps and disparities mostly to the disadvantage of women. In recent years, there has been growing response by government and some private actors to promote the welfare of women. Notable among these policies is the establishment of an entire government ministry responsible for women’s welfare. While this is laudable, it is important to complement it with policies that encourage females’ engagement in economic activities. This will help boost their bargaining power in the household.

While the findings of the study are closely relevant to public policy and can be generalized, there were some limitations that deserve to be mentioned. First, the variables used in the analysis may be limited in their measurement. For instance, the women’s bargaining power variables were measured using two dimensions; decision making for health and household purchases. We believe that broader measures of women’s bargaining power may improve our study. The study is also limited by the lack of panel data that could track women and observe their fertility choices over time. Causality is difficult to infer based on cross-sectional data alone. Future studies could consider these limitations in improving upon our work.

## Conclusion

This paper explores how exposure to child mortality influence fertility decisions among women in Ghana. We also examined the heterogeneous relationship between exposure to child mortality risks, women’s bargaining power and fertility preferences. The results confirm a strong relationship between exposure to child mortality risks and fertility preferences. We found that women who are more exposed to child mortality risks are more likely to prefer more children. The interaction effects suggest that, the effect of risk exposure worked mainly through women with relatively less bargaining power in the household. Such women are likely to prefer more children in the face of child mortality risk. The findings demonstrate that family planning efforts should look beyond access and utilization of contraceptives. Reducing childhood mortality risks and improving intra-household bargaining power will be a step in the right direction.

## Data Availability

The DHS used for this study is available upon request. Data can be obtained from the DHS program.

## Abbreviations

CSM: Contraceptive Social Marketing
DHS: Demographic and Health Survey
NHIS: National Health Insurance Scheme
SDGs: Sustainable Development Goals
TFR: Total Fertility Rate
